# The Contributing Role of Family, School, and Peer Supportive Relationships in Protecting the Mental Wellbeing of Children and Adolescents

**DOI:** 10.1007/s12310-022-09502-9

**Published:** 2022-02-06

**Authors:** Nadia Butler, Zara Quigg, Rebecca Bates, Lisa Jones, Emma Ashworth, Steve Gowland, Margaret Jones

**Affiliations:** 1 Public Health Institute, Liverpool John Moores University, 3rd Floor Exchange Station, Tithebarn Street, Liverpool, L2 2QP UK; 2 School of Psychology, Liverpool John Moores University, Liverpool, UK; 3 Sefton Council, Sefton, UK

**Keywords:** Mental wellbeing, Resilience, Family support, School support, Peer support, Children and adolescents

## Abstract

Globally, mental disorders are the leading cause of disability in children and adolescents. Previous research has demonstrated that supportive relationships are a key protective factor against poor mental health in children, particularly amongst those who have experienced adversity. However, fewer studies have examined the relative impact of different types of supportive relationships. The current study examined the association between level of family adult support, school adult support, and school peer support and mental wellbeing in a sample of children (age 8–15 years, *N* = 2,074) from schools in the UK. All three sources of support were independently associated with mental wellbeing. Analyses demonstrated a graded relationship between the number of sources of support and the odds of low mental wellbeing (LMWB), reflecting a cumulative protective effect. While all three sources of support were best, it was not vital, and analyses demonstrated a protective effect of school sources of support on LMWB amongst children with low family support. Peer support was found to be particularly important, with prevalence of LMWB similar amongst children who had high peer support (but low family and school adult support), and those who had high family and school adult support, (but low peer support), indicating that high peer support has an equivalent impact of two other protective factors. Findings from the study highlight the crucial context schools provide in fostering positive peer relationships and supportive teacher–student relationships to promote mental health and resilience for all children, including both those with and without supportive home environments.

## Introduction

Mental disorders are the leading cause of disability in young people globally, and at least one in five young people are estimated to experience a mental disorder in a given year (Patel et al., 2007; World Health Organization, 2012; Gore et al., 2011). In 2017, one in eight children in England had a mental disorder, and there has been an increase in the prevalence of disorders over the past two decades, particularly emotional disorders (NHS Digital, 2018; Patalay & Fitzsimons, 2017; Pitchforth et al., 2018). Untreated mental illness in childhood has profound implications across numerous domains of functioning and is associated with poor academic achievement (McLeod et al., 2012; Deighton et al., 2018), behavioural problems (Vorhaus & Vorhaus, 2012), school exclusion and truancy (Ford et al., 2018; Wood et al., 2012; Egger et al., 2003), substance use (Kandel et al., 1997; Goodwin et al., 2004), violence (Elborgen & Johnson, 2009; Arseneault et al., 2000), and delinquency (Sibley et al., 2011). Substantial global evidence has demonstrated that the risk of experiencing mental health difficulties across the lifecourse is strongly associated with childhood socioeconomic disadvantage, abuse and neglect, and other adversities (Edwards et al., 2003; McLaughlin et al., 2012; Hughes et al., 2016, 2019; Raposo et al., 2014; Turner et al., 2006; Dube et al., 2001). Crucially, childhood mental illness is one of the most evidenced predictors of psychiatric disorders in adulthood, with over half of all mental health difficulties in adulthood having their onset before the age of 14 (Fombonne et al., 2001; Reef et al., 2011; World Health Organization Regional Office for Europe, 2018). Thus, understanding the factors that both promote positive mental health in childhood and protect against mental health difficulties in children with high levels of risk is of vital importance in order to help prevent the onset of difficulties at this potentially critical point for the development of disorders.

Multiple research studies have identified the availability of stable, supportive relationships during childhood as an important determinant of mental wellbeing in childhood (Chu et al., 2010; Morgan et al., 2007) and across the whole lifecourse (Hughes et al., 2018). Social support is proposed to positively impact wellbeing as both a promotive factor (a factor which is associated with increased resilience and healthy functioning regardless of the presence of risk) and a moderator or protective factor (a factor that directly buffers against negative outcomes in individuals with high levels of risk) (Beeble et al., 2009). Studies have shown a significant association between positive supportive relationships with parents and youth’s wellbeing and life satisfaction, underlining the importance of caring, supportive families in fostering all children’s social and emotional wellbeing, regardless of level of risk (Gilman & Huebner, 2003; Oberle et al., 2011). Furthermore, positive social relationships develop individual level capacities, such as social competencies and self-esteem, which are promotive factors and essential for good mental health across the lifecourse for all individuals (Bagwell et al., 1998; Hartup, 1989). Crucially, such supportive relationships protect against the toxic effects of extreme stress on development by providing high risk children with a safe space to recover and develop healthy stress response systems (National Scientific Council on the Developing Child, 2015). Retrospective studies with adults suggest that social support (e.g. trusted adult support and positive peer relationships) in childhood is associated with lower levels of mental illness in adulthood for both those with and without childhood adversity (Hughes et al., 2018). For example, warm, supportive parenting has been demonstrated to mitigate the impact of family financial difficulty on mental health difficulties in early and middle childhood, with high levels of supportive parent–child interaction a more important predictor of child development than measures of disadvantage (e.g. parental education or income) (Sylva et al., 2008; Kirby et al., 2020).

While supportive relationships typically begin in the family, and attachment to a primary caregiver is argued to have one of the most salient influences on a child’s development (Ainsworth & Bell, 1970; Bowlby, 1982; Harlow, 1958), findings from a longitudinal study have demonstrated that, beyond immediate family members, the most frequent positive role model, or trusted adult, in children’s lives is a teacher (Werner & Smith, 1989). Supportive relationships between students and their teachers have been found to positively influence children’s school engagement and achievement, social skills, problem-solving skills, and sense of purpose and autonomy (Morrison & Allen, 2007; Sharkey et al., 2008; Woolley & Bowen, 2007), all of which are promotive and protective factors. Furthermore, household and family dysfunction, in addition to exposure to more severe forms of abuse and neglect, is likely to disrupt parent–child relationships. Thus positive and supportive relationships with teachers may be even more crucial for children who experience adversity as they are less likely to have supportive relationships within the family environment (Levendosky et al., 2002; Morton & Browne, 1998; Werner, 2005; Zimrin, 1986; Hughes et al., 2018; Heard-Garris et al., 2018). Previous research has shown that high levels of support from any trusted adult in childhood, regardless of relationship, halves the prevalence of low mental wellbeing amongst adults who experienced high levels of childhood abuse and adversity, compared to those who experienced adversity but no such adult support (Bellis et al., 2017).

School peers can also be an important contributor to resilience, developing social competencies, building self-esteem, and providing a source of emotional and practical support (Hartup & Stevens, 1999). Friendships characterized by high social support and acceptance are associated with lower levels of mental health difficulties and behavioural problems (Rothon et al., 2011; McPherson et al., 2014). Evidence also demonstrates that peer relationships moderate the association between family adversity and child maladjustment (Criss et al., 2002; Schwartz et al., 2000; Malindi & Machenjedze, 2012). However, research is currently limited on the impact of such relationships on child mental health outcomes (Sharkey et al., 2008; Bellis et al., 2018). In addition to teacher and peer relationships moderating associations between family adversity and child maladjustment in at-risk children, emerging evidence suggests that positive relationships may function in an additive manner, with social support from peers, teachers, and other trusted adults reinforcing pre-existing positive developmental pathways in children with positive family relationships (Criss et al., 2002). Thus, substantial debate in the field exists regarding both the relative importance of different sources of support (e.g. family, teacher, and peer) on a range of child developmental factors (Laible & Thompson, 2007; Collins et al., 2000; Harris, 1995; Criss et al., 2002), whether support functions in an additive manner, and whether it is a promotive and/or protective factor (Criss et al., 2002).

### The Current Study

A large evidence base demonstrates the association between supportive relationships and mental health in children and adults, particularly amongst those who have experienced adversity; however, fewer studies have explored the relative impact of different types of supportive relationships. Thus, the current study aims to contribute to the existing evidence base by:

1. Determining whether different sources of social support, including family adult support, school adult support, and school peer support, are associated with mental wellbeing in children and adolescents;
2. Exploring whether the number of sources of support available is associated with mental wellbeing; and,
3. Examining whether school sources of support (i.e. adult support and peer support) function as promotive or protective factors against poor mental wellbeing in children and adolescents with and without family adult support.

## Methods

### Study Design and Procedure

The current study used a cross-sectional non-probability sampling design. All schools in a borough of the North West of England were contacted by the public health lead from the borough council^1^ and invited to take part in an online survey measuring levels of pupils’ mental wellbeing and resilience. Principals who wished for their school to take part in the study acted as gatekeepers, and upon return of their signed consent form to the research team were provided with a letter, a detailed information sheet and both opt-in and opt-out parent/caregiver consent forms. Principals were asked to use their own discretion to decide on the method of consent most appropriate to the year groups they planned on implementing the survey with. Schools were also provided with standard text about the aims and reasons for the study and a link to a website with study information. This information about the study could be provided to parents if schools chose not to provide them with an opt-in/opt-out option via a school website, newsletter, email, or letters home. Principals of participating schools were asked to provide written consent in loco parentis for children whose parents did not contact them to opt-out of the study or if the principal chose not to send out opt-out forms.

Child friendly participant information sheets were provided at the beginning of the survey to participating children, with separate age-appropriate information sheets provided for primary and secondary school children. Implied assent from children was taken on commencement of the survey. Participating children completed a developmentally appropriate, anonymous, online questionnaire on school computers on their own, on a whole class basis, supervised by a school teacher. Non-participating students were assigned other appropriate work to complete by the teacher. There were years of focus in which principals were encouraged to administer the surveys (Year 4 [age 8–9 years], Year 7 [11–12 years], and Year 9 [13–14 years] students^2^), but they were free to administer the surveys to students of any year group (but no younger than Year 3 [age 7–8 years]^3^). In secondary schools, where students attend courses with multiple teachers, principals were asked to ensure that the survey was implemented during just one subject course in order to avoid duplicating responses and students answering the survey in multiple courses. Ethical approval for the study was granted from Liverpool John Moores University Research Ethics Committee and the study was also reviewed and passed by the borough’s public engagement and consultation panel.

### Sample

Data were collected from 2074 children from 22 primary (ages 8–11 years, *n* = 1,280) and 5 secondary (age 11–15 years, *n* = 794) schools across a borough in North West England. The total number of students in participating schools was 12,153; thus, the study sample represented 17.1% of all students from participating schools.^4^ Information on the number of students who opted out was unavailable, nor was information available on the total number of students in each year group of participating schools; thus, calculating an exact response rate by participating year group was not possible. The mean age of participants was 10.4 years (range 8–15 years, SD = 2.02). Approximately equal numbers of males (51.4%, *n* = 1066) and females (48.6%, *n* = 1008) participated in the survey.

### Measures

Final measures selected for inclusion were drawn from Public Health England guidance on measuring mental health and wellbeing in students, and measures used have been demonstrated to be suitable for use by children and are considered feasible in a school setting (i.e. not too long or requiring specific equipment) (Public Health England, 2016). Extensive consultation with school representatives, including principals, mental health leads, school nurses, and a community mental health team was undertaken about the content and methodology of the measures to ensure questions supported wider trauma-informed practices being implemented across the area.

#### Sources of Support

The Student Resilience Survey is a 47-item measure comprising 12 subscales measuring students’ perceptions of their individual characteristics as well as protective factors embedded in the environment (Lereya et al., 2016). Three subscales measuring supportive relationships in the family, school, and peer groups were used in the current study. The family and school support scales refer to an adult at home or school, respectively, and each includes four items (e.g. at home/at school there is an adult who… is interested in my school work; believes that I will be a success). The peer support scale included 10 items, drawn from the original 12-item scale,^5^ referring to aspects of support and friendship with other children at school (e.g. are there children at your school who would… tell you you’re their friend; help you if you hurt yourself). Items were scored on a 5-point scale (1 = *never* to 5 = *always*). Total scores on each scale were dichotomized to represent high and low levels of support, with low support defined as > 1 standard deviation below the mean (low family support scores < 15.12 [*M* = 17.54, SD = 2.42], low school support scores < 12.58 [*M* = 16.11, SD = 3.53], and low peer support scores < 31.20 [*M* = 39.41, SD = 8.21]). The Student Resilience Survey has previously been validated in a sample of UK primary and secondary school children, and good internal consistency was found for all subscales (family support subscale, *a* = 0.80; school support subscale, *a* = 0.89; peer support subscale, *a* = 0.93) (Lereya et al., 2016).

#### Mental Wellbeing

Mental wellbeing was measured in the current study using age-appropriate standardized measures: the Warwick Edinburgh Mental Wellbeing Scale (WEMWBS) (for secondary school participants aged 11 + years) and the Stirling Children’s Wellbeing Scale (SCWBS) (for primary school participants aged 8–11 years). The SCWBS and WEMWBS measure similar components of wellbeing using a positive and holistic approach, and there is a significant strong positive correlation between the two measures indicating construct validity (Liddle & Carter, 2013). WEMWBS is a 14-item scale measuring mental wellbeing in the general population which has been validated with English and Scottish children aged 13 years and above, with findings demonstrating the scale measures one strong underlying factor and has good internal consistency (*a* = 0.87) (Clarke et al., 2010). While it has not been validated with younger children, it has been used in previous research with children aged 11 years and above (Public Health England, 2016). WEMWBS was used with all participating secondary school children (aged 11 + years) in the current study. The scale consists of a series of statements about feelings and thoughts (e.g. I’ve been feeling useful; I’ve been feeling loved) and participants are asked to select the answer to each statement which best describes their experience over the past two weeks. Responses are scored on a 5-point scale (1 = *none of the time* to 5 = *all of the time*), resulting in a minimum score of 14 and maximum score of 70. Total scores on WEMWBS were dichotomized to define low mental wellbeing as > 1 standard deviation (12.09) below the mean (48.14); thus, low mental wellbeing was operationalized as scores < 36.05.

SCWBS is a 12-item scale measuring emotional and psychological wellbeing suitable for children aged 8–15 years. SCWBS was used in the current study with all participating primary school children (aged 8–11 years). It has previously been validated in Scottish children and findings demonstrated the scale had good internal reliability (*a* = 0.85), construct validity and external reliability, and appeared to be a robust measure of wellbeing in younger children (Liddle & Carter, 2015). The scale consists of two subscales comprising 6 items each, which provide scores for positive emotional state (e.g. I’ve been feeling calm) and positive outlook (e.g. I think lots of people care about me), the sum of which can be calculated to determine a total wellbeing score. Participants are asked to select the response to each statement which best describes their thoughts and feelings over the past couple of weeks. Responses are scored on a 5-point scale (1 = *never* to 5 = *all of the time*), resulting in a minimum score of 12 and maximum score of 60. Total scores on SCWBS were dichotomized to define low mental wellbeing as > 1 standard deviation (9.02) below the mean (45.82), thus low mental wellbeing was operationalized as scores < 36.83.

A new mental wellbeing variable (1 = *low mental wellbeing*, 0 = *high mental wellbeing*) for all participants was created by combining relevant cut-off scores on WEMWBS (< 36.05) for secondary school children or SCWBS (< 36.83) for primary school children.

### Statistical Analysis

Data were analysed with SPSS v.26. Analyses employed chi square for independence with continuity correction for initial bivariate examination of associations between mental wellbeing and sources of support and gender. An independent samples t-test was used to examine the association between mental wellbeing and age. Multivariate modelling used binary logistic regression to examine the independent relationships between family adult, school adult, and school peer support, and gender and age, with mental wellbeing. As the assessed relationships are clustered within schools, the nature of the data is inherently hierarchical and characteristics of the school may influence the results. To control for this, multivariate models were tested using linear mixed modelling to consider cluster random effects. The intraclass correlation coefficient (Bickel, 2007) was 0.175, indicating that approximately 17% of the variability in mental wellbeing was due to the school the participant attended and thus a multilevel analysis was justified (Kreft & de Leeuw, 1998). The effects of sources of support on mental wellbeing were assessed with random effects specified in these analyses, which consisted of adjusting errors for clustering at school level with a random intercept model. Modelled estimates for prevalence of low mental wellbeing were calculated for different levels and combinations of sources of support using an estimated marginal means function to adjust estimates for age and gender (IBM, 2017).

## Results

Overall, over one quarter (26.5%, *n* = 502) of participants had low mental wellbeing (LMWB). There was a significant association between mental wellbeing, and age and gender. Those with LMWB were older (*M* = 11.5 years, SD = 2.1) than those who did not have LMWB (*M* = 9.9 years, SD = 1.8, *p* < 0.001). A higher proportion of females had LMWB compared to males (Table 1).

**Table 1:** Bivariate and unadjusted relationships between sources of support and gender, and low mental wellbeing

	Low mental wellbeing						
	*n*	%	*χ*^2^	*p*	*OR*	95% CIs	
						*LL*	*UL*
*Gender*							
Male	222	23.0			Ref		
Female	280	30.2	12.106	< 0.001	1.45	1.18	1.78
*Family adult support*							
High	339	21.6			Ref		
Low	163	50.8	115.256	< 0.001	3.75	2.92	4.82
*School adult support*							
High	316	20.3			Ref		
Low	186	55.2	171.205	< 0.001	4.83	3.77	6.19
*Peer adult support*							
High	313	19.7			Ref		
Low	189	61.6	228.800	< 0.001	6.51	5.02	8.46

*OR* odds ratio, *95% CIs* 95% confidence intervals, *LL* lower limit, *UL* upper limit, *Ref.* reference category

### Sources of Support and Mental Wellbeing

Approximately one-sixth of participants had a low level of family adult support (FAS; 17.1%, *n* = 354), school adult support (SAS; 18.8%, *n* = 389), and school peer support (SPS; 17.9%, *n* = 372). There was a significant association between LMWB and levels of support across all three categories (FAS, SAS, and SPS) (Table 1). In multivariate analysis, when all forms of support, gender, and age were included in the model, all variables remained independently associated with LMWB (Table 2). Low levels of each type of support were significantly associated with increased odds of LMWB (Table 2). Female students had significantly higher odds of LMWB compared to males, independent of level of different sources of support, and the odds of LMWB increased as age increased (Table 2).

**Table 2:** Adjusted relationships between sources of support, gender and age, and low mental wellbeing

	Low mental wellbeing			
	*AOR*	95% CIs		*p*
		*LL*	*UL*	
Low family adult support	2.69	2.00	3.61	< 0.001
Low school adult support	2.08	1.54	2.80	< 0.001
Low school peer support	6.64	5.16	8.54	< 0.001
Female gender^a^	1.96	1.44	2.67	< 0.001
Age	1.35	1.23	1.49	< 0.001

*AOR *adjusted odds ratio, *95% CIs* 95% confidence intervals, *LL* lower limit, *UL* upper limit.

^a^Reference category = male

### Level of Support and Mental Wellbeing

Overall, almost two-thirds (63.6%, *n* = 1319) of participants had a high level of support from all three sources. Approximately one quarter (23.2%, *n* = 479) of participants had a high level of support from two sources, 9.3% (*n* = 192) had a high level of support from one source, and 4.1% (*n* = 84) of participants had no high level of support from any of the three sources.

**Fig. 1:**
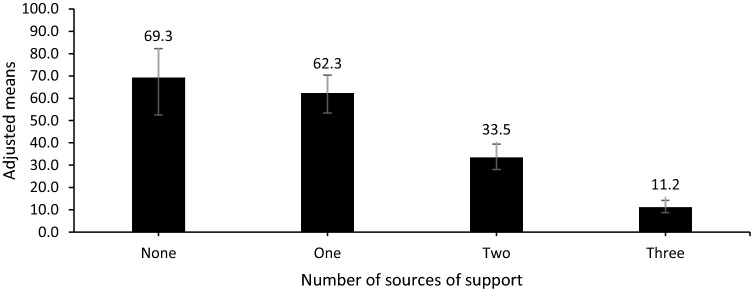
Adjusted proportion (95% CIs) of children with low mental wellbeing by number of high level sources of support

In multivariate analysis after adjusting for age and gender, there was a significant association between the number of sources and mental wellbeing, with a graded relationship between the number of high level sources of support and odds of LMWB (Table 3). The odds of LMWB were over 17, 13, and four times higher for those with no high level sources of support, one source or two sources, respectively, compared to those with the high level of support from all three sources (Table 3). Modelled prevalence estimates reflect these findings, with the lowest prevalence of LMWB amongst those with all three sources of support at a high level, and increasing prevalence of LMWB as number of sources of support decreased (Fig. 1).

**Table 3:** Adjusted relationships between number of high level sources of support, gender and age, and low mental wellbeing

	Low mental wellbeing			
	AOR	95% CIs		*p*
		LL	UL	
*Number of high level sources of support*				
Three				Ref
Two	4.01	3.18	5.15	< 0.001
One	13.15	8.35	20.70	< 0.001
None	17.96	9.92	32.56	< 0.001
*Gender*^*a*^				
Female	1.84	1.35	2.49	< 0.001
Age	1.30	1.20	1.42	< 0.001

*AOR* adjusted odds ratio, *95% CIs* 95% confidence intervals, *LL* lower limit, *UL* upper limit, *Ref.* reference category

^a^Reference category male

### School Sources of Support in Children and Adolescents With and Without Family Support

In bivariate analysis, there was a strong relationship between level of FAS and SAS (χ^2^ = 147.037, *p* < 0.001); level of FAS and SPS (χ^2^ = 123.339, *p* < 0.001); and level of SAS and SPS (χ^2^ = 169.236, *p* < 0.001). Due to these strong relationships and to meet study objectives of exploring the association between mental wellbeing and different sources of support, for multivariate analyses, the individual support variables were combined to create a new overarching sources of support variable. Participants were then categorized into the following groups: *high overall support* (high FAS, SAS, and SPS*), low overall support (*low FAS, SAS, and SPS*)*, *high FAS only* (low SAS and SPS), *high SPS only* (low FAS and SAS), *high SAS only* (low SPS and FAS)*, low FAS only* (high SPS and SAS), *low SPS only* (high FAS and SAS), and *low SAS only* (high FAS and SPS)*.*

In multivariate analyses, after accounting for the effects of age and gender, the combined support variable was significantly associated with mental wellbeing. Compared to those with high overall support, LMWB was two, three, and eight times higher for those with low school adult only, low family adult support only, and low peer support only, respectively, and seven times higher for those with high peer support only (Table 4). However, the combination of low peer support with low family and/or school adult support was associated with the highest increase in odds of LMWB (Table 4). Those with low overall support or with high family support only were over 18 times more likely to have LMWB compared to those with high overall support, while those with high school adult support only were over 20 times more likely to have LMWB (Table 4).

**Table 4:** Adjusted relationships between level of different sources of support, gender and age, and low mental wellbeing

Family adult support	School peer support	School adult support	Low mental wellbeing			
			AOR	95% CIs		*p*
				LL	UL	
High	High	High	Ref			
High	High	Low	2.44	1.73	3.43	< 0.001
High	Low	High	8.45	6.17	11.57	< 0.001
High	Low	Low	18.25	9.20	36.22	< 0.001
Low	High	High	3.42	2.47	4.73	< 0.001
Low	High	Low	7.54	4.17	13.64	< 0.001
Low	Low	High	20.14	10.93	37.11	< 0.001
Low	Low	Low	18.69	10.24	34.12	< 0.001
Female gender^a^			1.97	1.43	2.70	< 0.001
Age			1.35	1.22	1.49	< 0.001

*AOR* adjusted odds ratio, *95% CIs* 95% confidence intervals, *LL* lower limit, *UL* upper limit, *Ref* reference category

^a^Reference category = male

**Fig. 2:**
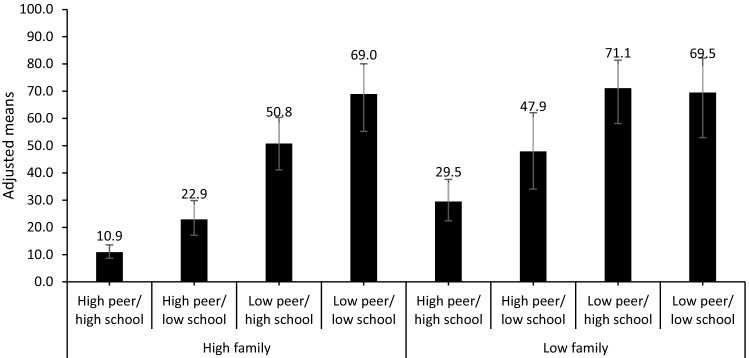
Adjusted proportion (95% CIs) of children with low mental wellbeing by level of family adult support and school adult and peer support

Modelled prevalence estimates reflect these findings with the lowest prevalence of LMWB amongst those with high overall support, with only 10.9% experiencing LMWB (Fig. 2). Prevalence of LMWB increased amongst those with low school adult support only and amongst those with low family support only to 22.9% and 29.5%, respectively (Fig. 2). Amongst those with high peer support only, and those with high school adult support only, prevalence of LMWB increased to 47.9% and 50.8%, respectively (Fig. 2). The highest prevalence of LMWB was amongst those with a combination of low peer support and low adult support (either family and/or school), with approximately 70% of individuals experiencing LMWB (low SPS and SAS group (69.0%), as well as the low SPS and FAS group (71.1%), and the low overall support group (69.5%); Fig. 2).

## Discussion

The purpose of the current study was to examine the association between different sources of support and mental wellbeing in children and adolescents. Findings indicated that family adult support, school adult support, and school peer support were all independently associated with mental wellbeing in children and adolescents. This suggests that all three sources of support are protective factors for wellbeing. Findings from the current study are consistent with previous research that demonstrates the association between supportive relationships and mental wellbeing (Hughes et al., 2018; Hartup, 1989). However, much of the previous literature focuses on the protective effect of having access to any one trusted adult in childhood (Bellis et al., 2017; Whitehead et al., 2019). Our study shows that school adult support and family adult support are independently associated with LMWB and function in an additive manner. Thus, having access to an adult both at home and in school provides a greater protective effect against LMWB than either one alone. Findings showed that amongst children with high peer support, when levels of either school or family support were low, prevalence of LMWB was approximately the same at 22.9% and 29.5%, respectively. When both family and school adult support were low, prevalence of LMWB rose to 47.9%. This is consistent with previous research in schools, which has shown that teachers and parents have independent impacts on student engagement and achievement (Brewster et al., 2007; Hughes & Kwok, 2007; Sylva et al., 2008). A study of European American school students by Wentzel (1998) found that the roles of teachers and parents worked in an additive way to promote student functioning and provided unique contributions to children’s level of engagement, with parental support associated with students’ academic goal orientations, whereas teacher support was related to interest in class and pursuit of goals. Thus safe, secure, and supportive home and school environments are both required for children and adolescents to develop and thrive (World Health Organization Regional Office for Europe, 2018).

While all sources of support were independently associated with mental wellbeing, findings from our study showed having all three sources of support was associated with the lowest prevalence of LMWB. Our analysis also demonstrated a graded relationship between the number of sources of support and the prevalence of LMWB in children and adolescents. Specifically, the prevalence of LMWB tripled when sources of support reduced from three to two and doubled when the number of sources of support reduced from two to one or none. This exponential relationship between sources of support and LMWB reflects a cumulative protection effect. Many models on cumulative risk exist and research has shown that the number of risk factors a child has is often more important than the nature of the risk factors in likelihood of poor outcomes (Ashworth & Humphrey, 2020). Our study demonstrates that protective factors such as supportive relationships may similarly provide a cumulative protective effect on mental wellbeing. Evidence for a cumulative protective effect has been found in previous studies of other outcomes such as youth violence (Stoddard et al., 2013), delinquency (Yoshikawa, 1994), and drug and alcohol use (Ostaszewski & Zimmerman, 2006).

While our study found that having all three sources of support was best and resulted in the lowest prevalence of LMWB, it was not vital, and analyses demonstrated a protective effect of school sources of support on LMWB amongst children with low levels of family support. While prevalence of LMWB was only 10.9% amongst children with high family, school, and peer support, amongst children with low family support, the prevalence of LMWB was 29.5% if school and peer support was high, but 69.5% if school and peer support was low. Further, if peer support and family support was high, prevalence of LMWB was 22.9%, suggesting that peer support plus the addition of one supportive adult, either at home or in school, has an approximate equivalent protective effect. This has important implications for children with low family support, where supportive adult relationships at school can compensate and protect against negative outcomes and is in line with previous studies of the protective impact of any type of adult support (Bellis et al., 2017). Poor family support and relationships have been associated with increased risk of child abuse and neglect, which is a positive predictor for poorer outcomes across the lifecourse, including mental health problems (Stith et al., 2009; Lindert et al., 2014; Chandon et al., 2019; Alm et al., 2020; Hughes et al., 2020; Butler et al., 2020). This highlights that the protective effect of school sources of support against LMWB could be crucial for children with low family support. However, evidence shows that adversity in childhood can lead to behavioural problems in school and is linked with increased likelihood of leaving school early, either voluntarily or through exclusion (Fry et al., 2018; Hardcastle et al., 2018; Jimenez et al., 2015). Furthermore, mental health problems are associated with disruptive behaviour, truancy, and low academic achievement, which further increases the risk of exclusion from school (NHS Digital, 2018; Paget et al., 2018). Thus, those potentially most at risk of exclusion are also those who might most need school sources of support to reduce risk of LMWB (and other negative outcomes). This suggests that a trauma-informed approach in schools could be beneficial, where staff are trained to understand how adversity can affect student learning and behaviour, and address this by providing alternative sources of support, such as peer and teacher relationships, to develop resilience and overcome adversity, rather than taking traditional punitive approaches to misbehaviour.

Study findings also suggested that peer support might be the most important protective factor against low mental wellbeing. After adjusting for age and gender, and controlling for family support and school adult support, children with low peer support were over six times more likely to experience low mental wellbeing. Furthermore, although we generally found a cumulative protection effect, where an increased number of sources of support was associated with an incremental decrease in prevalence of LMWB, when we examined the specific combinations of sources of support the importance of peer support was highlighted. The prevalence of LMWB was similar amongst children who had both low family and school support but high peer support (47.9%) to the prevalence in children who have high family and school support but low peer support (50.8%). This indicates that high peer support has an equivalent impact of two other protective factors: family support and school adult support, on mental wellbeing. This finding is in line with other studies, which have found that positive peer relationships can serve as protective factors for children at risk due to family adversity (Criss et al., 2002; Schwartz et al., 2000).

One possible explanation for these findings is that different types of relationships may meet different needs, thus why all three sources of support is best (Oldfield et al., 2016; Wentzel, 1998). However, in at-risk children where needs are not being met in a particular relationship context (e.g. at home), children may find other relationships (e.g. amongst peers) which meet these needs (Price, 1996). Evidence suggests that peer relationships are important developmental factors in shaping young people’s attitudes, behaviour, and identity, and developing self-esteem and social competence (Rubin, 1990; Reitz et al., 2014). Thus, positive peer relationships may function as a ‘remedial’ socialization context in at-risk children to develop skills not learned at home (Criss et al., 2002; Price, 1996). Furthermore, positive peer role models may function as a type of behavioural intervention for children who have been exposed to negative experiences in the home (Criss et al., 2002). For example, children exposed to harsh parenting and corporal punishment are at risk of developing externalizing behaviours and inappropriate ways of communicating and behaving with others (Lorber et al., 2011; Mendez et al., 2017). Positive peer role models may act as buffers and counteract and modify this behaviour learnt at home by teaching the child more prosocial means of interacting with others (Busching & Krahe, 2020). This would suggest that school-based peer support interventions would be effective in developing such relationships, strengthening resilience, and protecting against mental health problems. Peer support interventions are particularly suited to a school setting and involve students providing support or education to other young people in their school, often a younger year group. Such interventions are versatile in nature and can include peer mentoring, peer mediation, peer counselling, befriending, and buddying and focus on a range of issues such as bullying, violence, mental health, and school transitions (King & Fazel, 2019). While data suggest that most English primary and secondary schools use some kind of formal peer support scheme (Houlston et al., 2009), further research is needed to determine the effectiveness of peer-led interventions on mental health outcomes in school settings (King & Fazel, 2019).

The findings in the current study should be interpreted in light of a number of limitations. While analysis demonstrated associations between different sources of support and mental wellbeing, the cross-sectional study design prevents determination of the direction of the relationship. Thus, it cannot be said whether children with mental health issues have more difficulties forming relationships with family, teachers, and peers, or even whether they simply perceive these relationships as less supportive compared to children without such difficulties, or whether children with less support across a number of domains are at increased risk of low mental wellbeing. Data on deprivation and other child and school level factors, which may be potential mechanisms for the association between sources of support and wellbeing, were not included in the current study; however, variation between schools was accounted for in our mixed modelling analysis. Further, while we controlled for age and gender in the analysis, this may have obscured potential differences in the impact of different sources of support on mental wellbeing depending on the developmental stage of the child (e.g. middle childhood vs. adolescence) and gender. The current sample did not lend itself to exploring these factors and is an important area for future research. Two items were removed from the Peer Support Scale following stakeholder consultation and in line with trauma-informed practices across the region, and this may have had an impact on the subscale’s psychometric properties and resultant findings. Finally, the current study used a convenience sample and no data was available regarding the number and characteristics of those who did not participate in the study. Therefore, the study could have inadvertently excluded children with poorer supportive relationships (poor parent/teacher relationship making them more likely to be withdrawn from the study), or children who may have already been excluded from school who may have poor relationships and/or more likely to have low mental wellbeing.

## Conclusion

Mental health is a key target in the United Nations Sustainable Development Goals (SDGs) and the need to look beyond the health sector to tackle the causes of poor mental health is increasingly being recognized (United Nations, 2018). Findings from the current study highlight the crucial role schools can play in fostering positive relationships amongst students and supportive teacher–student relationships to promote mental health and resilience for all children, including both those with and without supportive home environments. Considering that not only do children and adolescents account for a large proportion of the burden of global mental disease, but that poor mental health in childhood is one of the most evidenced-based predictors of psychiatric disease as an adult, preventing childhood mental health problems could be key to achieving the SDG Goal 3 (ensuring health lives and promoting wellbeing for all at all ages). While many individual schools are now beginning to adopt a trauma-informed approach and/or implement interventions such as peer support programmes, for this to be scalable to a national level it may require legislation and allocation of funding to schools that are designated specifically for mental health provision. The UK government is currently considering a Bill (Schools (Mental Health and Wellbeing) Bill) to amend the Education Act 2002, which will make provision for state-maintained schools to promote the mental health and wellbeing of their pupils alongside academic attainment (UK Parliament, 2020). Such legislation would ensure schools are formally recognized as more than just places of learning and supported in their role in developing other crucial competencies necessary for children and adolescents to develop and thrive. This emphasis on student mental health and wellbeing may be crucial now more than ever, and findings from the current study take on particular relevance in light of current circumstances, with schools across the world returning following disruptions and closures due to the COVID-19 pandemic. Much of the focus by stakeholders in the education sector has been on ‘catching up’ with missed education and curriculum; however, findings from our study would suggest that promoting mental health through re-establishing positive, trusting relationships amongst peers and between students and teachers should be as important a priority going forward.

## Data Availability

Data used in the current study are available upon reasonable request.

## Footnotes

1 Local government responsible for a wide range of statutory and non-statutory services, including for example, education and social care services.

2 Equivalent to USA 3rd, 6th and 8th Grades.

3 Equivalent to USA 2nd Grade.

4 The total number of students at participating schools includes students from Year 1 and Year 2, who were ineligible to participate in the study.

5 After consulting with relevant stakeholders, there was consensus that the peer support scale, which contained 12 items, could potentially be distressing for children without such peer support. Stakeholders acknowledged the importance of measuring levels of support across the three domains, however from a trauma-informed perspective, they requested the subscale was shortened to lessen the potential impact on students with fewer friends. The removed items included: “are there students at your school who would… (1) pick you on their team, and (2) tell you secrets”. These questions were decided on by stakeholder consensus because the majority argued that not all children are good at activities involving teams and this may be why they are not selected, and telling secrets was considered something that should not be encouraged.
